# Forecasting the United State Dollar(USD)/Bangladeshi Taka (BDT) exchange rate with deep learning models: Inclusion of macroeconomic factors influencing the currency exchange rates

**DOI:** 10.1371/journal.pone.0279602

**Published:** 2023-02-07

**Authors:** Amrijit Biswas, Iftekhar Ahmed Uday, K. M. Rahat, Mst. Shapna Akter, M. R. C. Mahdy

**Affiliations:** Department of Electrical and Computer Engineering, North South University, Bashundhara, Dhaka, Bangladesh; Hanyang University, KOREA, REPUBLIC OF

## Abstract

Forecasting a currency exchange rate is one of the most challenging tasks nowadays. Due to government monetary policy and some uncertain factors, such as political stability, it becomes difficult to correctly forecast the currency exchange rate. Previously, many investigations have been done to forecast the exchange rate of the United State Dollar(USD)/Bangladeshi Taka(BDT) using statistical time series models, machine learning models, and neural network models. But none of the previous methods considered the underlying macroeconomic factors of the two countries, such as GDP, import/export, government revenue, etc., for forecasting the USD/BDT exchange rate. We have included various time-sensitive macroeconomic features directly impacting the USD/BDT exchange rate to address this issue. These features will create a new dimension for researchers to predict and forecast the USD/BDT exchange rate. We have used various types of models for predicting and forecasting the USD/BDT exchange rate and found that Among all our models, Time Distributed MLP provides the best performance with an RMSE of 0.1984. Finally, we have proposed a pipeline for forecasting the USD/BDT exchange rate, which reduced the RMSE of Time Distributed MLP to 0.1900 and has proven effective in reducing the error of all our models.

## Introduction

Ever since the world economy got heavily dependent on international trade, buying goods and services from a country requires an individual or an organization to buy them in the accepted local currency of that country. For example, if an organization purchases goods from Bangladesh, that entity must pay in Bangladeshi Taka (BDT) despite having United States Dollars (USD). The currency exchange rate plays a vital role in this transaction. That organization can exchange US Dollars with Bangladeshi Taka depending on the exchange rates set by the central bank of Bangladesh. So the Exchange rate means the value of one nation’s currency in terms of another. As exchange rates became a crucial element in international trade, predicting currency exchange rates became a demand and a challenge for businesses and individuals involved in the FOREX (foreign exchange market). In the earlier days, economists tried to evaluate their mathematical exchange-rate models using the horse race approach, where they saw which model performed better in predicting the actual values of the exchange rate. Machine learning techniques have added a new dimension by making devices self-learner. Machine learning algorithms are capable of doing complex calculations faster and capable of making decisions more accurately. For that reason, predicting a currency exchange rate has shifted from manual accounting to machine learning algorithms, which have proven much more efficient and accurate than previous approaches. Over the last 15 years, the unanimity on the determinants of currency exchange rate movements has further broken down. The actual reason for the currency exchange movements can be explained partly by the world economy and the development of new theories of exchange rate determination. The work done in this paper has focused on the perspectives of the world economy in the exchange rate movements of two economies by taking into account the driving forces of the currency supply and demand in the global market. Bangladeshi Taka (BDT) follows the floating exchange rate regime where the value of Bangladeshi Taka (BDT) depends on the global supply and demand of Bangladeshi currency [1]. In macroeconomics policy, a floating currency exchange rate is a type of exchange rate field in where the value of a currency fluctuates in response to foreign exchange market events [2]. Though the USD/BDT exchange rate prediction was made before using time series models, machine learning, and neural network models, much more inaccuracy still needs to be addressed in most previous research works. But in the existing studies on the USD/BDT exchange rate, there was no inclusion of the underlying macroeconomic factors, which are independent variables and drive the currency supply and demand in the economy. All the existing research just used the USD/BDT exchange rate pattern to train the models but ignored the factors that can directly impact the exchange rate. None of them trained their models using the underlying variables or features. The challenge is to improve the results even by a small margin to mitigate investment risk management and higher returns while maintaining an investment portfolio.

The approach of this study for forecasting the USD/BDT exchange rates stands on the combination of macroeconomic theory with deep learning and machine learning algorithms, where the macroeconomic factors drive the exchange rate volatility. This study has considered the driving macroeconomic factors of a floating currency since Bangladesh maintains a floating currency regime. The exchange rate of the USD/BDT depends on the driving forces of supply and demand in the FOREX market. Some macroeconomic factors of the United States government influence the supply and demand of the FOREX market. Those macroeconomic factors of the United States economy have been included as the determinants of exchange rate fluctuations in the global FOREX market. Again, some macroeconomic factors of Bangladesh have been included in this study that also drives the monetary authority of Bangladesh to set exchange rates for the USD/BDT. Finally, we have combined the macroeconomic factors from the United States and Bangladesh, as all these factors influence the USD/BDT exchange rate. After that, we trained our models using this new dataset to extract the relation between these factors and the USD/BDT exchange rate. This new approach of considering the economic perspectives in exchange rate prediction has significantly improved results. This new approach is much more reliable and accurate because models are now trained using legit factors that directly impact the USD/BDT exchange rate. Previously, researchers have trained their models by using the USD/BDT exchange rate pattern only. Those approaches were much more statistical than considering the underlying reasons behind the fluctuation of the USD/BDT exchange rate. Even with neural networks such as Artificial Neural Networks (ANN), researchers only used the pattern/trend of the USD/BDT exchange rate to train their models. This USD/BDT exchange rate forecasting method needs more information to train a model and to predict accurately. To address this issue, we have included macroeconomic features while training our models so that the features can be provided enough information to our model to learn precisely about the USD/BDT exchange rate. The other researchers have yet to make this novel approach for forecasting the USD/BDT exchange. Since macroeconomic factors have been considered in this study, with a significant amount of economic data, it’s even possible to forecast exchange rates in a longer timeframe. Overall, this novel empirical approach will create a new dimension for the researchers to factor in their insights and develop models for better prediction and reduce investors’ risk in the FOREX market. Reducing investors’ investing risk in the FOREX market will significantly increase investment. For a developing country such as Bangladesh, it is essential to increase investment to improve its economy. Therefore the main concept of this research can be described by these points- (i) Previously, researchers have forecasted the USD/BDT exchange rate by using the pattern of the USD/BDT exchange rate only. Those approaches were much more statistical than considering the underlying factors behind the change in the USD/BDT exchange rate. (ii) To address this issue. First, We have sorted out the reasons behind the change in the USD/BDT exchange rate from an economic perspective. (iii) Secondly, we extracted the models that can be used for forecasting the USD/BDT exchange rate. (iv) Then, we combined the idea of economics with neural networks and machine learning models in our research. We have trained our models using 16 macroeconomic factors that impact the change of the USD/BDT exchange rate and found a better result than other existing research. (v) Finally, We have proposed a pipeline for improving the result further.

## Related works

Before starting the implementation of our research work, we explored a lot of related research works in the field of machine learning, neural networks, and economics. Some notable research works related to our USD/BDT currency exchange rate forecasting have been mentioned below-

A study by Saeed A, Awan RU, Sial MH, Sher F. [3] has analyzed the determinants of the exchange rate between USD and PKR within the framework of the monetary approach. They have taken stock of money, foreign exchange reserves, and total debts of Pakistan relative to the USA as the dummy variables and took them as the determinants of the exchange rate. They have applied the ARDL approach to the co-integration and error correction model. The results confirmed that stock of money, debt, and foreign exchange reserves are significant determinants of the exchange rate. Yao J, Tan CL [4] have provided empirical evidence that a neural network model is applicable for predicting the exchange rate. The authors have fed time-series data, moving averages into the model to determine the underlying rules of exchange rate movements between different currencies. Their work further showed that foreign currency’s dynamic supply and demand factors made it difficult to predict the foreign exchange rates effectively. This aligns with the fundamentals of economics, which are that prices increase when the demand is greater than the supply and decrease when the demand is less than the supply. Since FOREX is a currency market, this fundamental aspect of economics stands true.

Using data mining techniques, Carbureanu M [5] have tried to predict the Romanian Leu/Euro. In that study, they mentioned that political, economic, and social events in a given timeline influence the supply and demand of a currency and have an instant effect on the currency exchange rate. Since there is no control over political and social events, our study looked into the economic factors that strongly impact currency exchange rates.

Ramasamy R, Abar SK. [6] have used the yearly currency rates of three countries along with their macroeconomic variables, such as relative interest rates, to verify their impact on exchange rate movements. They used the bootstrapping technique to increase the sample size and run regressions to study the effect. According to their study, macroeconomic variables such as tax rate, the balance of payments, inflation rate, interest rates, and other factors randomly influence the exchange rates. However, these macroeconomic variables might be unstable depending on the state of the economy within a country.

Twin A [7] has stated a few macroeconomic factors that influence the currency exchange rates: inflation rate, interest rate, current account deficit, public debt, terms of trade, and strong economic performance. According to the exchange rate regime, the floating exchange rate is influenced by the market’s driving forces of supply and demand, where the fixed/pegged exchange rate is determined and maintained by the government or central bank [8]. We have found some interesting nature of exchange rate movement and exposure for Bangladesh [9].

Zwanger S [10] have conducted a study to outline the effects of modern exchange-rate theory on the exchange rate movements of Chili and the United States. Since the Chilean Peso (CLP) is pegged to the USD, they have considered the independent variables of the monetary policy interest rate, money supply, and inflation rates. However, they have mentioned that these independent variables might lose their explanatory ability when economic conditions change or in the case of switching in the foreign exchange rate policy dictated by the central bank.

A study by Cushman DO [11] has tested the risk effects of real exchange rates on U.S. bilateral trade flows during the floating period using a few of the new and previously used risk measures. They have mentioned that other factors affect the calculation of exchange rates in third-world countries. According to the law of the fixed price model, the prices of goods in different countries which are traded internationally are identical in the perfect market.

A study by Akhtar MA, Hilton RS [12] has found that uncertainty of exchange rate significantly impacts the imports and exports of Germany and the USA. They have established a negative relationship between the volatility in the exchange rate and the volume of international trade.

In their study, Broil U, Eckwert B [13] have mentioned that developing countries have insignificant access to international capital. Thus, the domestic inflation rate is connected with fixed/pegged exchange rates. A study by Kemal MA [14] has mentioned that in the case of Bangladesh, the exchange rate volatility is positively related to exports and negatively to imports. Furthermore, they have concluded that currency devaluation occurs by balancing the trade deficit.

Bouraoui T, Phisuthtiwatcharavong A [15] have conducted a study on THB/USD exchange rate where they explained how the Thai central bank intervenes in response to certain concerns and shocks in the managed floating regime. In this scenario, the study outlines six important factors that influence the THB/USD exchange rate: interest rates differential, manufacturing production index, terms of trade, monetary base, government debt, and international reserves.

Refenes AN, Zapranis A, Francis G [16] have conducted a study where they have proved neural networks could outperform the statistical forecasting techniques when the non-linearities apply in the dataset of stock indices. They have shown that using sensitivity analysis and neural networks can provide a rational explanation of their predictive behavior and model their environment more convincingly than regression models.

Rehman M, Khan GM, Mahmud SA [17] have used CGP and Recurrent Neural Network to predict the exchange rates between AUD and three other currencies. An approach of Recurrent Neuro-Evolution was taken to forecast the currency exchange rate. They have observed that the computational method outperformed other statistical methods due to the ability to select the best feature in real-time, feature selection flexibility, and effectively recognize patterns.

Islam MS, Hossain E [18] have predicted the exchange rates of major currency pairs using the GRU-LSTM hybrid network. They tested the results with the standalone GRU and standalone LSTM models and found that the hybrid model outperformed the standalone models. This provides us with the idea of using a hybrid model to predict the USD/BDT exchange rate.

Pandey TN, Jagadev AK, Dehuri S, Cho SB [19] have reviewed the neural network and statistical models to predict the exchange rate and also proposed a machine that identifies the shortcomings of both the neural network and statistical models. They have found that multilayer neural networks had BAYESIAN learning predictive accuracy performed better than neural networks using backpropagation learning.

Rout M, Majhi B, Majhi R, Panda G [20] have forecasted the exchange rates using an adaptive autoregressive moving average (ARMA) model with differential evolution-based training. They have compared the ARMA-DE model with other competitive models and found that it outperformed other models for the long and short time predictions. The performance was measured based on the model’s training time and accuracy. Panda MM, Panda SN, Pattnaik PK [21] have used convolutional neural networks for multi-currency exchange rate prediction. They have proposed a model that can develop multivariate exchange rate information and use those features better. They have used the adaptive learning rate (ADAM) optimization technique to provide optimal weight for their proposed model.

Majhi R, Panda G, Sahoo G [22] have used low-complexity artificial neural network models for efficient exchange rate prediction. This study has developed two ANN models: functional link ANN (FLANN) and cascaded functional link ANN (CFLANN). The models involve nonlinear inputs and a simple ANN structure with one or two neurons. They have observed that CFLANN works better than FLANN having the least error.

Refenes AN, Azema-Barac M, Chen L, Karoussos S [23] have applied a multilayer perceptron network to predict the currency exchange rate and discussed the convergence issues related to network architecture.

Galeshchuk S, Mukherjee S [24] have stated that time series models and shallow neural networks result in acceptable estimates in the future prices for exchange rates but perform poorly at predicting the direction of change. On the other hand, machine learning classifiers trained on input features curated based on domain knowledge produce better results.

Damrongsakmethee T, Neagoe VE [25] have implemented a deep learning model with Long Short Memory (DLSTM) to predict the currency exchange rate of USD/THB and took financial inputs such as interest rate, gross domestic product rate (GDP), balance account, inflation rate, and balance of trade also a finite set of previous exchange rates. Wang J, Wang X, Li J, Wang H [26] have used the model of CNN-TLSTM for USD/CNY exchange rate prediction. So going forward with neural networks and deep learning models has been a credible approach for us.

Baffour, A. A., Feng, J., and Taylor, E. K [27] have integrated the GJR(Glosten Jagannathan and Runkle) model with Artificial Neural Networks (ANN) model for forecasting the volatility of five currency exchange rate. They have found a significant improvement by using the ANN-GJR hybrid model rather than using benchmark models. These findings influence us to use the CNN-LSTM hybrid model for forecasting the USD/BDT exchange rate.

In their research, Roy, S.S., Chopra, R., Lee, K.C., Spampinato, C. and Mohammadi-ivatlood, B. [28] have compared and analyzed the Random Forest, Gradient Boosting Model and Deep Neural Network for predicting the stock of South Korean companies. In their research, Random Forest performed better than other models, and possible reasons for Deep Neural Networks’ lower performance can be the relatively small data set and difficulties in data augmentation.

Bose, A., Hsu, C. H., Roy, S. S., Lee, K. C., Mohammadi-Ivatloo, B., and Abimannan, S. [29] have proposed a hybrid model by cascading Multivariate Adaptive Regression Splines(MARS) and Deep Neural Network(DNN) for predicting the closing price of the stock. They have found an accuracy of 92% while predicting the closing price of the exchange rate.

Our study has focused on the connection between the USD/BDT exchange rate through the underlying factors of exchange rate movement and proved the concept using different models. Researchers have yet to follow our approach for forecasting the exchange rate of USD/BDT in a dual floating currency setup. While doing the entire research project, we have followed some modeling, evaluating, statistical, and research methods for implementing our neural network models from here [30, 31].

## Materials and methods

### Loss function

We have used the Mean squared error (MSE) for all the models as the loss function. This loss function calculates the average squared difference between the actual and predicted values.

### Optimization function

After trying different optimizers, we have found that Adaptive Moment Estimation (Adam) [32] helped the models to converge faster. Therefore, we have used the Adam optimizer to optimize the learnable parameters of all the models.

### Hyperparameters optimization

First, We have tried grid search and random search hyperparameter optimization methods. Using grid search can be a time-consuming process where there are more hyperparameters. The random search may ignore some important combinations of hyperparameters. Also, Some of our hyperparameters were discrete values, and some were continuous. Grid and random search do not support discrete and continuous values simultaneously. Therefore, we have applied the Bayesian optimization method, which is more advanced than random and grid searches. Finally, manually hyperparameter values were set several times to make sure the best hyperparameters have been selected by comparing the results.

### Performance metrics

We have forecasted the USD/BDT exchange rate by using various models, and the outputs of those models are continuous values. To make sure how good or bad our models are, we have calculated the error of our model. The badness of a model is how much error is generated by that model while predicting the USD/BDT exchange rate. For measuring the error of our models, we have used Root Mean Square Error (RMSE) which is used widely as a performance metric for regression analysis, forecasting, climatology, and so on. RMSE is known as the Standard deviation of residuals. RMSE penalizes the error more than the Mean Absolute Error (MAE) and Mean Absolute Percentage Error (MAPE) by squaring the error. For that reason, we have selected RMSE as our primary performance measurement metric, as well as Mean Absolute Percentage Error(MAPE). Finally, to check how well our models fit the data, we have calculated the R-squire value (R2).

$$\begin{array}{c} RMSE=\sqrt{\frac{1}{N}\sum_{i=1}^{N}{\left(y_{i}-\hat{y_{i}}\right)}^{2}} \end{array}$$ (1)

$$\begin{array}{c} MAPE=\frac{1}{N}\sum_{i=1}^{N}\frac{|y_{i}-\hat{y_{i}}|}{y_{i}} \end{array}$$ (2)

$$\begin{array}{c} R^{2}=1-\frac{\sum_{i=1}^{N}{\left(y_{i}-\hat{y_{i}}\right)}^{2}}{\sum_{i=1}^{N}{\left(y_{i}-\bar{y}\right)}^{2}} \end{array}$$ (3)

Where *y*_*i*_ is the actual value of the USD/BDT exchange rate, ${\hat{y}}_{i}$ is the predicted value of the USD/BDT exchange rate, and N denotes the total number of predictions or actual values. The model is better when the RMSE and MAPE are lower, and the model is bad when the RMSE and MAPE are high. The value of R2 is between 0 to 1. The closer the value of R2 to 1, the better the model is.

### Dataset details

We have collected the USD/BDT exchange rate from 2000 to 2019 from the directory of Bangladesh Bank. Other records of different macroeconomic features have been collected from the website Trading Economics (https://tradingeconomics.com/) with a paid seven days trial subscription. Both the USD/BDT exchange rate and records of other macroeconomic factors have been collected simultaneously for the same time and merged to complete the dataset. The dataset contains a total of 5000 records of USD/BDT exchange rates. We have considered the parameter “Price” as the target and the rest of the 16 macroeconomic factors as our features for training the models. Thsoe macroeconomic factors are:

1. Gross Domestic Product (GDP) of USA
2. The inflation rate of the USA
3. Foreign reserve of USA
4. Exports of USA
5. Imports of USA
6. Money supply (M0) of USA
7. Money supply (M1) of USA
8. The interest rate of the USA
9. Government revenue of Bangladesh
10. Money supply (M0) of Bangladesh
11. Money Supply (M1) of Bangladesh
12. Balance of Trade of Bangladesh
13. Exports of Bangladesh
14. Imports of Bangladesh
15. GDP annual growth rate of Bangladesh
16. The inflation rate of Bangladesh

We have 16 continuous macroeconomic factors and are interested in predicting the exchange rate of USD/BDT using those macroeconomic factors/features. So, our main interest is to find out the relationship between the factors and the target. To find the relationship, we have used Pearson and Pearson (Table 1) and Spearman’s correlation (Table 2). Spearman’s correlation is most commonly used worldwide to find the correlation between feature and target. Sometimes Pearson correlation can be misinterpreted when the data are homogeneous. For that reason, we have considered Spearman’s rank-based correlation, which is much more precise than the Pearson correlation. However, we have calculated and compared Pearson’s and Spearman’s correlation but didn’t notice any notable change in values.

**Table 1:** Correlation (Pearson) between features(factors) and target.

Factors	Correlation value with USD/BDT exchange rate (target)
*Exports of USA*	0.96
*Imports of USA*	0.95
*Gross Domestic Product* (*GDP*) *of USA*	0.95
*Exports of Bangladesh*	0.93
*Imports of Bangladesh*	0.92
*Government revenue of Bangladesh*	0.88
*Money supply* (*M*0) *of USA*	0.88
*Money Supply* (*M*1) *of Bangladesh*	0.87
*Money supply* (*M*1) *of USA*	0.87
*Money supply* (*M*0) *of Bangladesh*	0.87
*Balance of Trade of Bangladesh*	0.81
*Foreign reserve of USA*	0.78
*GDP annual growth rate of Bangladesh*	0.71
*The inflation rate of Bangladesh*	0.34
*The inflation rate of the USA*	-0.28
*The interest rate of the USA*	-0.51

**Table 2:** Correlation (Spearman’s) between features(factors) and target.

Factors	Correlation value with USD/BDT exchange rate (target)
*Government revenue of Bangladesh*	0.95
*Gross Domestic Product* (*GDP*) *of USA*	0.95
*Money Supply* (*M*1) *of Bangladesh*	0.95
*Money supply* (*M*0) *of Bangladesh*	0.95
*Money supply* (*M*1) *of USA*	0.95
*Imports of Bangladesh*	0.94
*Exports of Bangladesh*	0.94
*Imports of USA*	0.93
*Exports of USA*	0.93
*Money supply* (*M*0) *of USA*	0.90
*Balance of Trade of Bangladesh*	0.87
*GDP annual growth rate of Bangladesh*	0.73
*Foreign reserve of USA*	0.71
*The inflation rate of Bangladesh*	0.20
*The inflation rate of the USA*	-0.33
*The interest rate of the USA*	-0.50

The correlation values represent the direction of the relationship and how related or important a factor is. A positive correlation value represents a positive relationship, and a negative correlation value denotes a negative relationship. Those factors with correlation values close to positive ones have a very strong positive relationship with the target. And the factors with correlation values close to negative ones have a very strong negative relationship with the target.

After observing the correlation values, it is clear that almost all factors strongly correlate with the target, which is the USD/BDT exchange rate. So, we can use those factors for forecasting the USD/BDT exchange rate. The correlation tables are given below, first the Pearson correlation followed by Spearman’s correlation-

### Splitting dataset

The dataset has sequentially split. The first 70% of data has been used for training, sequentially the following 20% data for validation, and the reset 10% for testing. This resulted in 3500 records for training, 1000 for validation, and 500 for testing.

### Data preprocessing

Initially, we had a total of 19 features. Then after checking NULL values, we found that three of our features have more than 80% NULL values. So, we have dropped those three features. After that, the Date column was converted into a date-time object and set as the index. Then we checked for duplicate values and found no duplicate values in our dataset. After that, we scaled our dataset using multiple scalers such as min-max scaler and standard scaler. We have used principle component analysis (PCA) for feature extraction. Finally, we have converted the data into a suitable size for neural networks by using a window size of 30 and a batch size of 32.

### Methodology

For each model, we have trained our models for 300 epochs by using the Adam optimizer and mean squared error as our loss function. We have also used model checkpoints and early stopping callbacks. The purpose of the model checkpoint is to monitor the validation loss of our model and save the best model. Early stopping will also monitor the value of validation loss. The patience has been set to 10. So if the validation loss does not decrease within ten epochs, the training process of our model will be stopped. After training our models, we saved our best models in HDF5 format.

#### LSTM

LSTM is a trendy model for time series forecasting. Due to the ability to process the entire sequence of data and the potential to learn long-term observation, this model has become a trending choice for researchers. LSTM can learn long-term patterns, such as yearly patterns, and the model can learn short-term patterns, such as monthly or weekly patterns.

Three parts comprise an LSTM network cell: a cell state, a hidden state, and three gates. Cell states are used to pass information through the network. Those are concerned with the entire input passed through so far. The input gate is used to add information to the cell states. The input gate regulates what values need to be added by using a sigmoid function. This acts like a filter for all the information starting from h(t-1) to x_t_. The hidden layer creates a vector of all possible values by using the tanh function. Finally, the regulated value is multiplied by the vector and added to the cell states. The forget gate removes information from the cell state to reduce redundancy. This gate takes two inputs: h (t-1) and x_t_. The following equations do the entire process-

$$\begin{array}{c} c_{t}=f_{t}*c_{t-1}+i_{t}*c_{t} \end{array}$$ (4)

$$\begin{array}{c} h_{t}=o_{t}*tanh\left(c^{t}\right) \end{array}$$ (5)

$$\begin{array}{c} i_{t}=\sigma \left(w_{i}\left[h_{t-1},x_{t}\right]+b_{i}\right) \end{array}$$ (6)

$$\begin{array}{c} f_{t}=\sigma \left(w_{f}\left[h_{t-1},x_{t}\right]+b_{f}\right) \end{array}$$ (7)

$$\begin{array}{c} o_{t}=\sigma \left(w_{o}\left[h_{t-1},x_{t}\right]+b_{o}\right) \end{array}$$ (8)

Where, i_t_, f_t_, o_t_, represents the input gate, forget gate and output gate and c_t_, h_t_, represents the activation vectors where w and b are weight matrix and bias.

As our dataset contains 16 features with a Date column. For that reason, we have used multivariate LSTM. For training our LSTM model, we have used a total no of 3500 featured records starting from 15th February 2000. By applying hyperparameter optimization, We have tuned the activation function of the dense layer, dropout ratio, and learning rate. For monitoring the progress, we have used the validation loss. We found that the softplus activation function performed better than all other activation functions. For the dropout ratio of 0.1 and a learning rate of 6e -6, our model suits the data better than any additional dropout ratio and learning rate value. Then we implemented the LSTM model by using those hyperparameters, and other hyperparameters were set as default.

#### Bi-LSTM

In LSTM, the input is passed to the output in a single direction. Which is known as the forward direction. But in Bi-LSTM, the flow of input to output is not unidirectional. In the Bi-LSTM model, two LSTM models are used. One is used for taking the input in the forward direction, and another LSTM is used in the backward direction. The sequence model Bi-LSTM can be more promising than LSTM as it provides more information to the network. The hidden layers of Bi-LSTM store two values. A matrix for calculating forward direction values and a transpose matrix for backward calculation. Finally, the output depends on the matrix calculated from the forward direction and the transpose matrix calculated from the backward direction. The working process of Bi-LSTM is given below-

$$\begin{array}{c} a_{h}^{t}=\sum_{n=i}^{L}x_{i}^{t}W_{th}+\sum_{h^{{}^{\prime }},t>0}^{H}b_{h^{{}^{\prime }}}^{t-1}W_{h^{{}^{\prime }}h} \end{array}$$ (9)

$$\begin{array}{c} a_{h}^{t}=\theta_{h}\left(a_{h}^{t}\right) \end{array}$$ (10)

Where L is the input unit for forward calculation and H is the number of hidden units.

For training our Bi-LSTM model, we have used the same number of records we used for LSTM. By applying hyperparameter optimization, We have tuned the activation function of the dense layer, dropout ratio, and learning rate. For monitoring the progress, we have used the validation loss. We found that the softplus activation function performed better than all other activation functions. For the dropout ratio of 0.1 and a learning rate of 4e -4, our model fits the data better than any other value of dropout ratio and learning rate. Then we implemented the Bi-LSTM model by using those hyperparameters, and other hyperparameters were set as default.

#### Stacked LSTM

A stacked LSTM model consists of multiple LSTM layers instead of a single hidden LSTM layer. Instead of passing a single value output, the LSTM layer provides sequence output to the next LSTM layer. So, the next LSTM layers getting input from a previous layer can create a more complex feature representation. This enables the Stacked LSTM model to learn from the input data from different perspectives at each timestamp. The equations used for updating the layers of Stacked-LSTM are given below-

$$\begin{array}{c} i_{l}^{t}=\sigma \left(W_{l}^{i}h_{l-1}^{t}+V_{l}^{i}h_{l}^{t-1}+b_{l}^{f}\right) \end{array}$$ (11)

$$\begin{array}{c} f_{l}^{t}=\sigma \left(W_{l}^{f}h_{l-1}^{t}+V_{l}^{f}h_{l}^{t-1}+b_{l}^{f}\right) \end{array}$$ (12)

$$\begin{array}{c} o_{l}^{t}=\sigma \left(W_{l}^{o}h_{l-1}^{t}+V_{l}^{o}h_{l}^{t-1}+b_{l}^{o}\right) \end{array}$$ (13)

$$\begin{array}{c} c_{l}^{t}=f_{l}^{t}*c_{l}^{t-1}+i_{l}^{t}*\text{tanh}\left(W_{l}^{c}h_{l-1}^{t}+V_{l}^{c}h_{l}^{t-1}+b_{l}^{c}\right) \end{array}$$ (14)

$$\begin{array}{c} h_{l}^{t}=o_{l}^{t}*\text{tanh}\left(c_{l}^{t}\right) \end{array}$$ (15)

Where l is the number of layers used for Stacking and $i_{l}^{t},f_{l}^{t},o_{l}^{t}$, represents the input gate, forget gate and output gate of l-th layer and $c_{l}^{t},h_{i}^{t}$, represents the activation vectors of l-th layer where w and b are weight matrix and bias.

In our research, we have used two layers of LSTM. We have used the same 3500 featured records for training the model starting from 15th February 2000. We have used the hyperparameters optimization technique for tuning the activation function, dropout ratio, and learning rate. Other hyperparameters are set as default. We have found that the activation function elu with a dropout ratio of 0.1 and a learning rate of 4e-4 provides the best fit for our dataset. While applying hyperparameter optimization, we monitored the validation loss of our model.

#### GRU

Gated Recurrent Unit (GRU) is also similar to the LSTM model. This model was introduced to solve the vanishing gradient problem of recurrent neural networks. Two gets were introduced to solve this issue. The update and reset gate. These two gates decide which information will go through the output. Transition functions of GRU applied in hidden units are-

$$\begin{array}{c} z_{t}=\sigma (W^{z}x_{t}+V^{z}h_{t-1}+\left(b^{z}\right) \end{array}$$ (16)

$$\begin{array}{c} r_{t}=\sigma (W^{r}x_{t}+V^{r}h_{t-1}+\left(b^{r}\right) \end{array}$$ (17)

$$\begin{array}{c} {\bar{h}}_{t}=\text{tanh}\left(W^{c}x_{t}+V^{c}\left(r_{t}.h_{t-1}\right)\right) \end{array}$$ (18)

$$\begin{array}{c} h_{t}=\left(1-z_{t}\right).h_{t-1}+z_{t}.{\bar{h}}_{t} \end{array}$$ (19)

Where z_t_ represents the update gate, r_t_ represents the reset gate, and h_t_ is the hidden state.

We have used the same number of records for training the GRU model. By using hyperparameters optimization, We have tuned the activation function of the dense layer, dropout ratio, and learning rate. For monitoring the progress, we have used the validation loss. The softplus activation function performed better than all other activation functions. For the dropout ratio of 0.1 and a learning rate of 4e -4, our model fits the data better than any other value of dropout ratio and learning rate. Finally, we implemented the GRU model by using those hyperparameters, and other hyperparameters were set as default.

#### ANN

The concept of the Artificial Neural Network(ANN) is derived from the working method of biological neurons. ANN has been used in a wide range due to its promising performance. Previously researchers have used this ANN model for forecasting the USD/BDT exchange rate, but they did not include any features. The working method of ANN starts with taking inputs, then the inputs are multiplied by the assigned weights and added together. Finally, this value goes through an activation function to get the final result. The working process is given below-

$$\begin{array}{c} s=\sum_{i=1}^{n}W_{i}*X_{i}+b \end{array}$$ (20)

$$\begin{array}{c} o=f(s) \end{array}$$ (21)

Where s is the weighted sum of inputs, f is the activation function, and o is the final output.

A total of 3500 records have been used to train our ANN model. By applying hyperparameters optimization, We have tuned the activation function, dropout ratio, and learning rate. For monitoring the progress, we have used the validation loss. The softplus activation function performed better than all other activation functions. Our model fits the data with a dropout ratio of 0.1 and a learning rate of 4e -4. Finally, we implemented our ANN model using those hyperparameter values, and other hyperparameters were set as default.

#### CNN

The convolutional neural network (CNN) is a popular deep learning algorithm that can assign importance to various aspects of any matrix and learn from them. The study by Koprinska I, Wu D, and Wang Z [33] showed the usage of CNN in time series forecasting. Using that concept, we have applied a 1D CNN model for USD/BDT forecasting. Moreover, we have a featured dataset, and CNN is a good model for extracting the pattern from features. All these reasons lead us to use the CNN model.

The CNN model has three layers. The convolutional layer, the pooling layer, and finally, the fully connected layer. The convolutional layer is responsible for extracting important features via the convolution process. Convolution is a process that involves the multiplication of input data with a set of weights. These are called filters or kernels. The pooling layer of CNN is used to reduce the spatial size of the convolved features. Doing this ensures that we use less computational power to process the data. We have used max pooling in our research. Max pooling extracts the maximum value of the area covered by the kernel. It can also suppress noise by discarding noisy activities. The pooled features are then passed to the fully connected layer as inputs. The weighted sum of the inputs in the fully connected layer is calculated. Then the final output is calculated by passing the weighted sum with some bias through an activation function. The equations for each step are given below-

$$\begin{array}{c} Z^{l}=h^{l-1}*W^{l} \end{array}$$ (22)

$$\begin{array}{c} h_{x,y}^{l}=max_{\left(i=0,..,s,j=0,..,s\right)}h_{\left(x+i)(y+j\right)}^{l-1} \end{array}$$ (23)

$$\begin{array}{c} z_{l}=\left(W_{l}*h_{l-1}\right)+b \end{array}$$ (24)

$$\begin{array}{c} o=f(z_{l}) \end{array}$$ (25)

Where z^l^ is the convolution operation, ${\text{h}}_{\text{x},\text{y}}^{\text{l}}$ denotes the max pooling, z_l_ represents the fully-connected layer, f is the activation function, and o is the output.

We have used 1D CNN with a total no of 48 filters with a kernel size of 3 for the convolutional operation. Casual padding has been used, and the value of stride is set to 1. After convolving, we used max-pooling with a pool size of 5. Then again, we have used hyperparameter optimization to find the best possible activation function for the fully connected layer and also to extract the best possible value of the learning rate. While selecting the best activation function and learning rate, we monitored the validation accuracy. After applying the hyperparameter optimization method, we have found that the sigmoid activation function and the value of learning rate 6e-3 suit our dataset better than any other combination of activation function and learning rate.

#### CNN-LSTM

A CNN-LSTM model contains a convolution operation for feature extraction on input data which then combines with the LSTM model for sequential predictions. CNN network is useful for extracting the most useful features from a set of inputs. LSTM can find the interdependence of time series data and the best forecasting model. In the work of Vidal A, Kristjanpoller W [34], they implemented CNN-LSTM to predict gold price volatility. Our dataset has features and times from 2000 to 2019 that affect the USD/BDT exchange rate value. The model CNN-LSTM potentially should be a very useful model for this dataset. As in our dataset, feature extraction and sequence prediction are very important. CNN-LSTM works sequentially. First, the input is passed to the CNN model. The output of the CNN model is then passed to the LSTM Model. The working procedure of CNN-LSTM is given below-

$$\begin{array}{c} Z^{l}=h^{l-1}*W^{l} \end{array}$$ (26)

$$\begin{array}{c} h_{x,y}^{l}=max_{\left(i=0,..,s,j=0,..,s\right)}h_{\left(x+i)(y+j\right)}^{l-1} \end{array}$$ (27)

$$\begin{array}{c} i_{t}=\sigma \left(w_{i}\left[h_{t-1}^{l},x_{t}\right]+b_{i}\right) \end{array}$$ (28)

$$\begin{array}{c} f_{t}=\sigma \left(w_{f}\left[h_{t-1}^{l},x_{t}\right]+b_{f}\right) \end{array}$$ (29)

$$\begin{array}{c} c_{t}=f_{t}*c_{t-1}+i_{t}*c_{t} \end{array}$$ (30)

$$\begin{array}{c} h_{t}=o_{t}*tanh\left(c^{t}\right) \end{array}$$ (31)

$$\begin{array}{c} o_{t}=\sigma \left(w_{o}\left[h_{t-1},x_{t}\right]+b_{o}\right) \end{array}$$ (32)

Where, Where, z^l^ is the convolution operation, ${\text{h}}_{\text{x},\text{y}}^{\text{l}}$ denotes the max pooling, i_t_, f_t_, o_t_, represents the input gate, forget gate and output gate and c_t_, h_t_, represents the activation vectors where w and b are weight matrix and bias.

In our CNN-LSTM model, the CNN layer has been organized into a convolutional layer and a max-pooling layer. In the first convolutional layer, we have used a total number of 100 filters with a kernel size of 2. Casual padding and a stride value of 1 have been used for this convolutional layer. After that, we used a max-pooling layer with a pool size of 5. Then the output of the CNN layer is passed to the LSTM layer. Like before, we have used hyperparameter optimization techniques for tuning filter size, activation function, and learning rate. While selecting the best combination of the filter size, activation function, and learning rate, we monitored the validation loss. We have found that filter size 2, relu activation function, and a learning rate of 4e-3 provide the best fit for our dataset.

#### Encoder-Decoder

Encoder-Decoder is an RNN architecture first used in 2014. This model has proven very effective in the case of sequence-to-sequence prediction. The Encoder-Decoder network is divided into three parts: the encoder part, the intermediate vector, and the decoder part.

The Encoder part consists of a stack of several RNN cells. Each cell accepts a single element of the input sequence, which extracts info from that input and passes it forward. The hidden states are computed using

$$\begin{array}{c} h_{t}=f\left(W^{hh}h_{t-1}+W^{hx}x_{t}\right) \end{array}$$ (33)

Here appropriate weight is applied to the previous hidden state, which is h(t-1), and the input vector x_t_, The final intermediate vector is calculated by using this formula. The vector uses all the input sequence information to help the decoder make accurate predictions. The intermediate vector acts as an initial hidden state for the decoder part.

The Decoder part is also made of several RNN cell stacks. Each cell predicts an output y_t_ at the t timestamp and creates its own hidden state. Decoder hidden states are computed by

$$\begin{array}{c} h_{t}=f\left(W^{hh}h_{t-1}\right) \end{array}$$ (34)

Like the Encoder part, the previous hidden state is used to compute the next. The output y_t_ at each timestamp t is given by

$$\begin{array}{c} y_{t}=softmax\left(W^{s}h_{t}\right) \end{array}$$ (35)

In the encoder part, we used LSTM as the RNN cell to encode the sequence. We have applied the hyperparameter optimization method for tuning hyperparameters. We have tried multiple activation functions and found that the activation function relu performs better than any other activation function. Then we calculated the intermediate vector. The vector uses all the input sequence information to help the decoder make accurate predictions. The intermediate vector acts as an initial hidden state for the decoder part. In the Decoder part, we have used the LSTM cell again for decoding. While trying multiple activation functions for the Decoder part, we have noticed that the relu activation function performs better than the others. In the dense layer, the activation function relu performs better with a learning rate of 1e-3. For selecting hyperparameters, in all the cases, we have monitored the validation loss.

#### Time distributed MLP

Time-distributed layers are very useful when working with time-series datasets. Time-distributed layers allow a single layer to be used for each input. Time-distributed layers are usually used with RNNs to keep one-to-one relation between input and output, which allows the dense nodes of the time-distributed layers to have the same weights and biases. The time-distributed vector is used to apply the same function at each timestep. The time-distributed layer analyzes each time step without considering other timesteps. Here we have applied a multilayer perceptron(MLP) with time-distributed layers. The working procedure of time-distributed MLP is mentioned below-

$$\begin{array}{c} h_{i}^{l}=\varphi \sum_{j}\left(\left(W_{ij}^{l}*x_{j}\right)+b_{i}^{l}\right) \end{array}$$ (36)

$$\begin{array}{c} y_{i}=\varphi \sum_{j}\left(\left(W_{ij}^{l}*h_{j}^{l}\right)+b_{i}^{l}\right) \end{array}$$ (37)

Where *φ* is the activation function, l is the number of layers, $h_{i}^{l}$ is the lth hidden layer, y_i_ is the output, $W_{ij}^{l}$ and $b_{i}^{l}$ are the weight matrix and bias of lth layer.

In our research, we have used four layers. We have tried different activation functions in each layer and monitored the validation loss. From the experiment, we have found that the activation function relu is better than the other activation functions. After that, we used the hyperparameter optimization technique to tune the activation function of the dense layer, dropout ratio, and learning rate. After applying the hyperparameter optimization and considering the validation loss, we have noticed that the combination of activation function relu, a dropout ratio of 0.1, and a learning rate of 2e3 provides the best performance.

#### SVM

Support Vector Machine, known as SVM, is one of the most popular machine learning models. Due to the ability to handle non-linear data, SVM is everywhere. It is a classification algorithm but can also be used for regression problems. Support Vector Machine works in this way-

$$\begin{array}{c} f\left(x,w\right)=\sum_{j=1}^{m}w_{j}g_{j}\left(x\right)+b \end{array}$$ (38)

Where g_j_(x) is the non-linear transformation; this non-linear transformation allows the separation of the data points by projecting them into a higher dimension. The kernel trick is a worldwide used non-linear transformation. In our research, we have used the same amount of data that we have used for other models after applying the hyperparameter optimization technique. We have found that a value of C = 1.0 and a value of epsilon = 0.2 suits better than other values. Other hyperparameters were set as default.

#### XGBoost

Extreme Gradient Boosting, known as XGBoost, is one of the ensemble techniques that has achieved great success in sequential decision tree machine learning algorithms. XGBoost uses most of the normal boosting algorithm function, but it is an advanced boosting algorithm implementation with regularization factors. XGBoost performed very well for tabular data, so it suited our work.

The main part of XGBoost is boosting. In this boosting process, trees are built one after another, and each tree targets to reduce the errors of its predecessor. This boosting is done in a few steps. An initial model F0 is defined, which will predict the target variable. Another model, h0 is fit to the residuals of previous steps. These two models are now combined to give F1, which will have a lower MSE than F0.

$$\begin{array}{c} F_{0}\left(x\right)+h_{0}\left(x\right)=F_{1}\left(x\right) \end{array}$$ (39)

We can now further improve performance by introducing a new model F2

$$\begin{array}{c} F_{1}\left(x\right)+h_{1}\left(x\right)=F_{2}\left(x\right) \end{array}$$ (40)

This can be repeated for ‘n’ iterations until we get desired residual minimization.

$$\begin{array}{c} F_{n-1}\left(x\right)+h_{n-1}\left(x\right)=F_{n}\left(x\right) \end{array}$$ (41)

There are some crucial hyperparameters to choose from when implementing XGBoost. Booster is the boosting algorithm. We have used the default option gbtree, for boosting. We have used the hyperparameter optimization technique for tuning other hyperparameters and monitored the validation loss. L1 and L2 regularizations are red alpha and red lambda. The greater their number, the less the model, will overfit. But it will also make the model miss more information. Here in our model, a value of 0.001 for alpha and 0.1 for lambda is more suitable for our dataset. We have used multiple values of the tree and gamma values and found that a value of 10 for the max depth and 0.005 for gamma provides better performance.

## Proposed pipeline for predicting USD/BDT exchange rate

**Fig 1:**
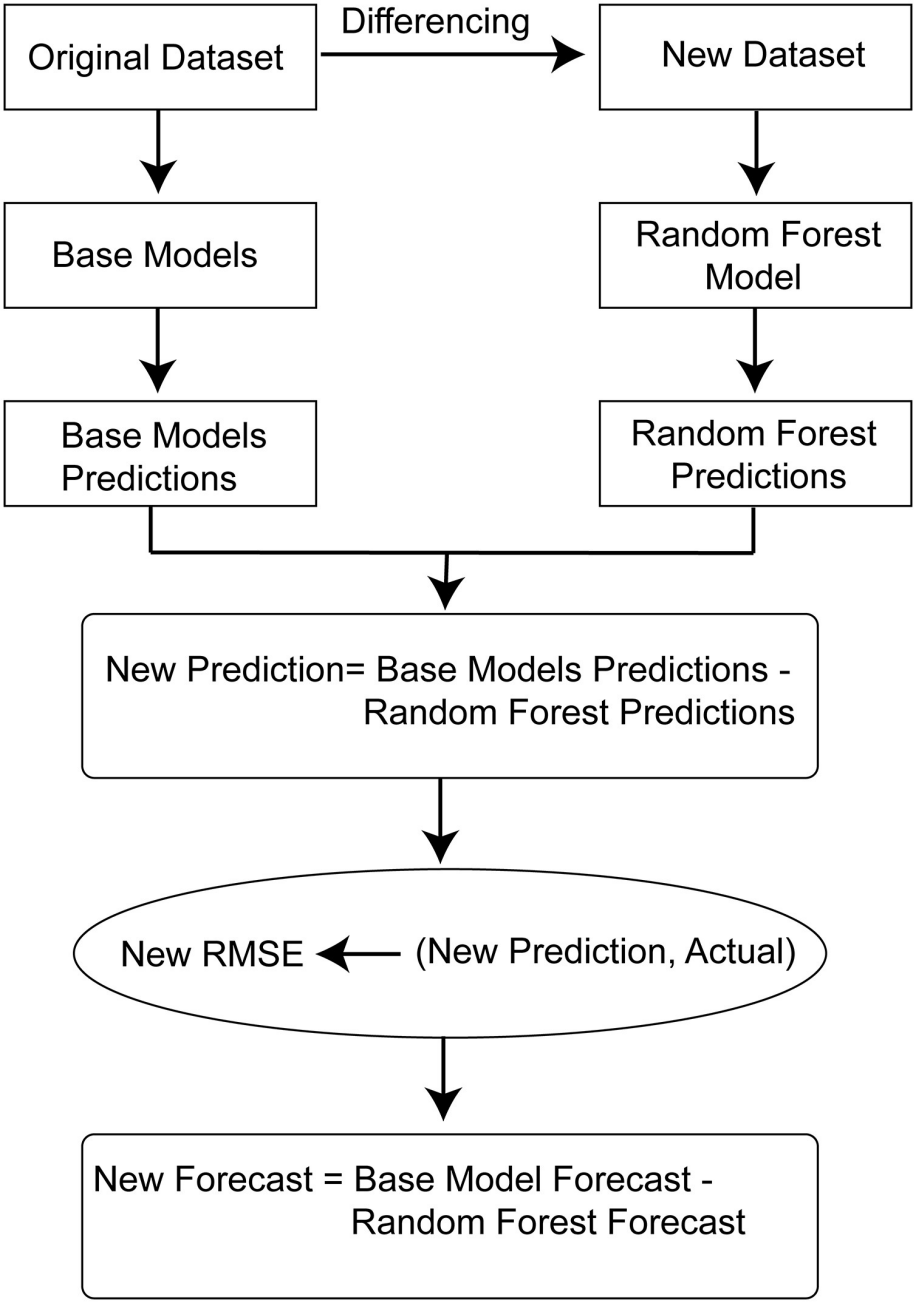
Proposed pipeline architecture for USD/BDT exchange rate prediction.

We have introduced a pipeline (Fig 1) for reducing the error of our baseline models where the baseline models are LSTM, Bi-LSTM, Stacked LSTM, GRU, ANN, CNN, CNN-LSTM, Encoder-Decoder, Time Distributed MLP, SVM, and XGBoost. We have used our baseline models to predict the USD/BDT exchange rate. We have designed the pipeline for all our baseline models further to reduce the USD/BDT exchange rate prediction error.

First, we have calculated the value of fluctuations for each feature by differencing the current value of a feature from the next value. After doing differencing, we have got a dataset derived from the original dataset. This new dataset contains the fluctuation data for each feature, including the target. This new dataset represents if the value of a feature fluctuates for a certain amount, then how much the price of the Dollar will fluctuate.

Therefore, this new dataset denotes the fluctuation in the USD/BDT exchange rate in terms of fluctuations in all features. Now we have trained a model using this new dataset to learn the fluctuation pattern of the USD/BDT exchange rate in terms of the fluctuation in other features. For that, a Random Forest regressor has been used. The fluctuation values of the features are the inputs of the Random Forest, and the fluctuation in USD/BDT is the target. The time of training, validation, and testing data for Random Forest has been kept the same as the time of our baseline models so that Random Forest can predict the fluctuations of the USD/BDT exchange rate for the same time as our baseline models have predicted the USD/BDT exchange rate. We have used a value of 7 for the hyperparameter max depth and 33 for the hyperparameter n_estimator. Other hyperparameters have been set as default. After training Random Forest using the new dataset, this Random Forest model can predict the USD/BDT exchange rate fluctuations. These predicted fluctuations are a kind of noise generated from the fluctuations of features. If we can deduct some fluctuations from our baseline model predictions, the predictions will be closer to the actual values. First, the predictions of USD/BDT exchange rates provided by the baseline models have been calculated. Also, the fluctuations in the USD/BDT exchange rate predicted by Random Forest for the same time used for USD/BDT exchange rates prediction by our baseline models have been calculated. The baseline models provide the USD/BDT exchange rates for a particular time, and the Random forest provides the predicted fluctuations in the USD/BDT exchange rate for that same time.

Finally, the fluctuations have been deducted from the predicted USD/BDT exchange rates by our baseline models. The predicted fluctuations by Random Forest can be positive or negative. Suppose a single value of the USD/BDT exchange rate, predicted by our baseline models, is greater than the actual USD/BDT exchange rate. In that case, the fluctuation value predicted by Random Forest for that single value of the USD/BDT exchange rate should be positive. In that case, subtracting the fluctuation from the predicted USD/BDT exchange rate will drive the predicted USD/BDT exchange rate closer to the actual USD/BDT exchange rate. Suppose the predicted USD/BDT exchange rate is less than the actual USD/BDT exchange rate. In that case, the predicted fluctuation value should be negative so that it becomes positive while doing subtraction. As a result, the predicted USD/BDT exchange rate gets closer to the actual exchange rate.

The error generates when there is a gap between the predicted and actual values. The main concept of our proposed pipeline is to make the predicted USD/BDT exchange rate closer to the actual exchange rate so that the error minimizes. But the problem is, let’s say, for example, for the first ten values, the predicted values are greater than the actual values. The predicted values for the next five values are less than the actual ones. Now, if we subtract a small fixed value from all the predicted values, the first ten predicted values will get closer to the actual values because those values were greater than the actual values. But the next five values will generate more error than the previous because those five values were less than the actual values, and subtracting a small value will create more distance from the actual values. Adding a small value will make the last five prediction values closer to the actual values, but again it will generate more distance for the first ten predicted values. So it is clear that adding or subtracting a small discrete value from the prediction value will not ensure that all the predicted values will get closer to the actual value. So it cannot ensure that the overall error will be reduced. Moreover, the gap between the predicted value and actual value is not equal and unidirectional everywhere, meaning predicted values are not either greater or less than actual values in all the situations. So choosing the small discrete value is also very critical. To address this issue, the small value we want to add or subtract will not be a fixed discrete value. Rather than considering a single small discrete value, we will consider different small values for different data points. Those different small values will be the predictions of Random Forest that we have introduced as predicted fluctuations. As we have mentioned earlier, the Random Forest model has been trained by using the features’ fluctuations, and the Random Forest’s prediction is the fluctuation in the USD/BDT exchange rate. That means the predictions of Random Forest describe how much the price of USD/BDT can fluctuate. So the USD/BDT exchange rate fluctuation will differ for every data point. These predicted fluctuation values can be interpreted as unwanted movements in the USD/BDT exchange rate. Those fluctuation values can be both positive and negative. Now, if we deduct the fluctuation values from the predicted values, there is a chance that the predicted values will get closer to the actual values. Because now we are not using a small discrete value for all the data points. Each small value (fluctuation) is dedicated to a single data point as specific small values are predicted for specific data points by Random Forest. The unidirectional problem has been solved because the predicted small values(fluctuations) of Random Forest can be both positive and negative. So if the predicted small value (fluctuation) is positive, the predicted USD/BDT exchange rate will be reduced after subtracting. Again, if the predicted small value (fluctuation) is negative, then the USD/BDT exchange rate will be increased after subtracting. However, it is impossible to predict each fluctuation value correctly. In our experiment, we found an improvement in RMSE for all our models while applying this proposed pipeline, indicating that most of the fluctuation predictions were correct. Therefore, after subtracting the fluctuations from the predicted USD/BDT exchange rates, the predicted USD/BDT exchange rates got closer to the actual price.

## Results

### Predicted price of USD/BDT

**Fig 2:**
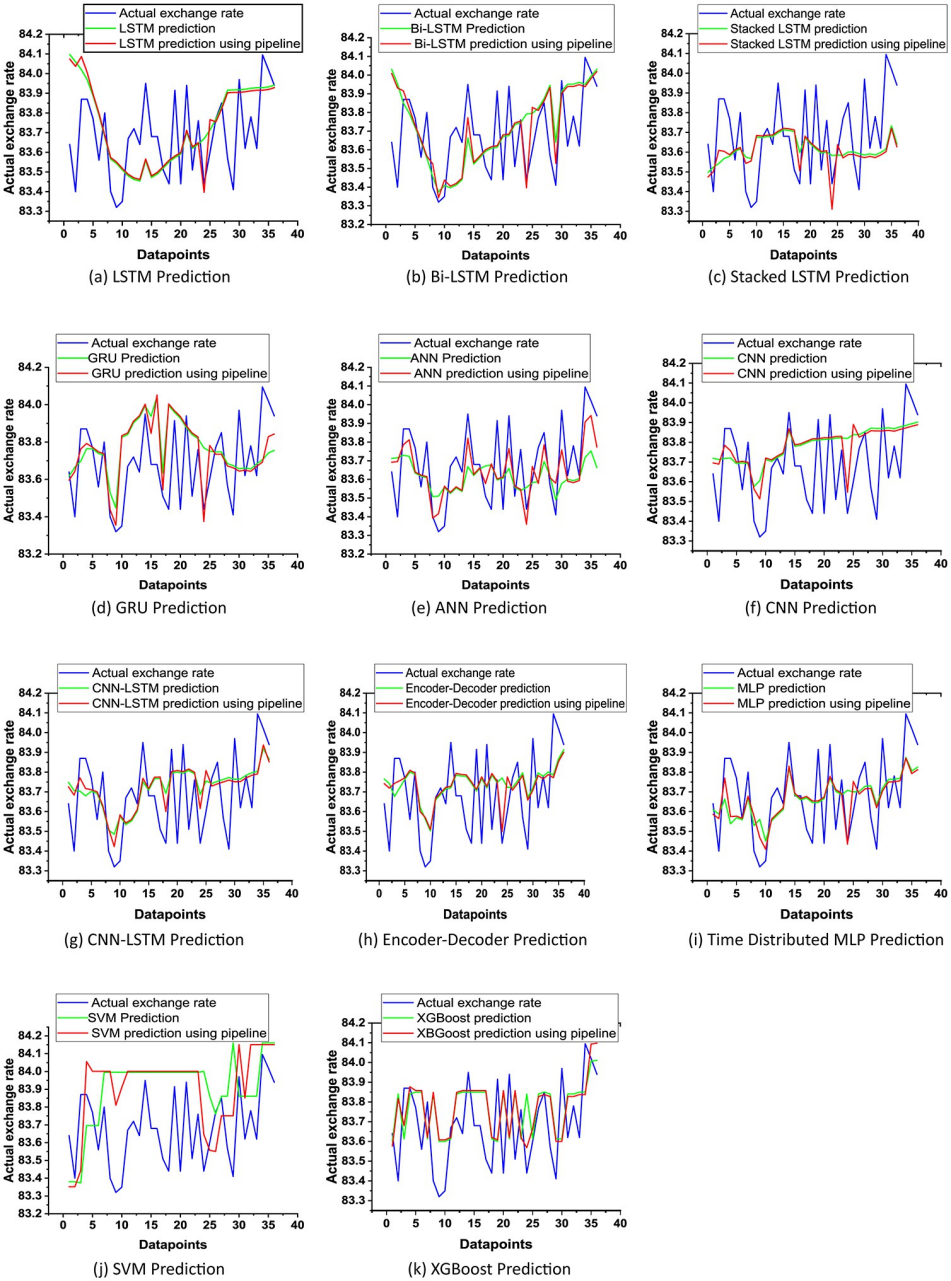
Plotting actual vs. predicted USD/BDT exchange rate by using the base models and also applying proposed pipeline. (a) LSTM, (b) Bi-LSTM, (c) Stacked LSTM, (d) GRU, (e) ANN, (f) CNN, (g) CNN-LSTM, (h) Encoder-Decoder, (i) Time Distributed MLP,(j) SVM, (k) XGBoost.

We have predicted the USD/BDT exchange rate using all our models and calculated the error of our models (Table 3) by using RMSE and MAPE. Most of the researchers have used the RMSE for evaluating their models. For that reason, the RMSE has been considered the main evaluation metric. To check the fitting of our models, we have considered the R2 score. Then we plotted the actual value of the USD/BDT exchange rate price vs. the predicted price (Fig 2). After that, we applied our proposed pipeline to our models to check the effectiveness of the proposed pipeline. Finally, we have compared our results with other research.

**Table 3:** Evaluating all the models’ prediction.

Models	RMSE	MAPE	R2
*LSTM*	0.2526	0.00244	0.9016
*Bi* − *LSTM*	0.2493	0.00242	0.9156
*Stacked* − *LSTM*	0.2239	0.00221	0.9288
*GRU*	0.2786	0.00269	0.8744
*ANN*	0.2196	0.00220	0.9355
*CNN*	0.2244	0.00223	0.9245
*CNN* − *LSTM*	0.2059	0.00214	0.9410
*Encoder* − *Decoder*	0.2257	0.00224	0.9228
*Time Distributed MLP*	0.1984	0.00205	0.9563
*SVM*	0.412	0.00432	0.7980
*XGBoost*	0.2467	0.00255	0.9189

Plotting actual vs. predicted USD/BDT exchange rate:

While plotting the predictions of our models vs. the actual USD/BDT exchange rate (Fig 2), it’s visualized that our models succeeded in generalizing the upward and downward trend of the exchange rate. The actual value of the exchange rate is between 83.3 BDT to 84.1 BDT, where we have made our predictions. This part of the dataset is the roughest part of the entire dataset, where the exchange rate fluctuation is much more random and has no visible pattern. The prediction could be even better if we chose the time duration where the fluctuation of USD/BDT is less and have some upward or downward trend.

### Forecasted price of USD/BDT

**Fig 3:**
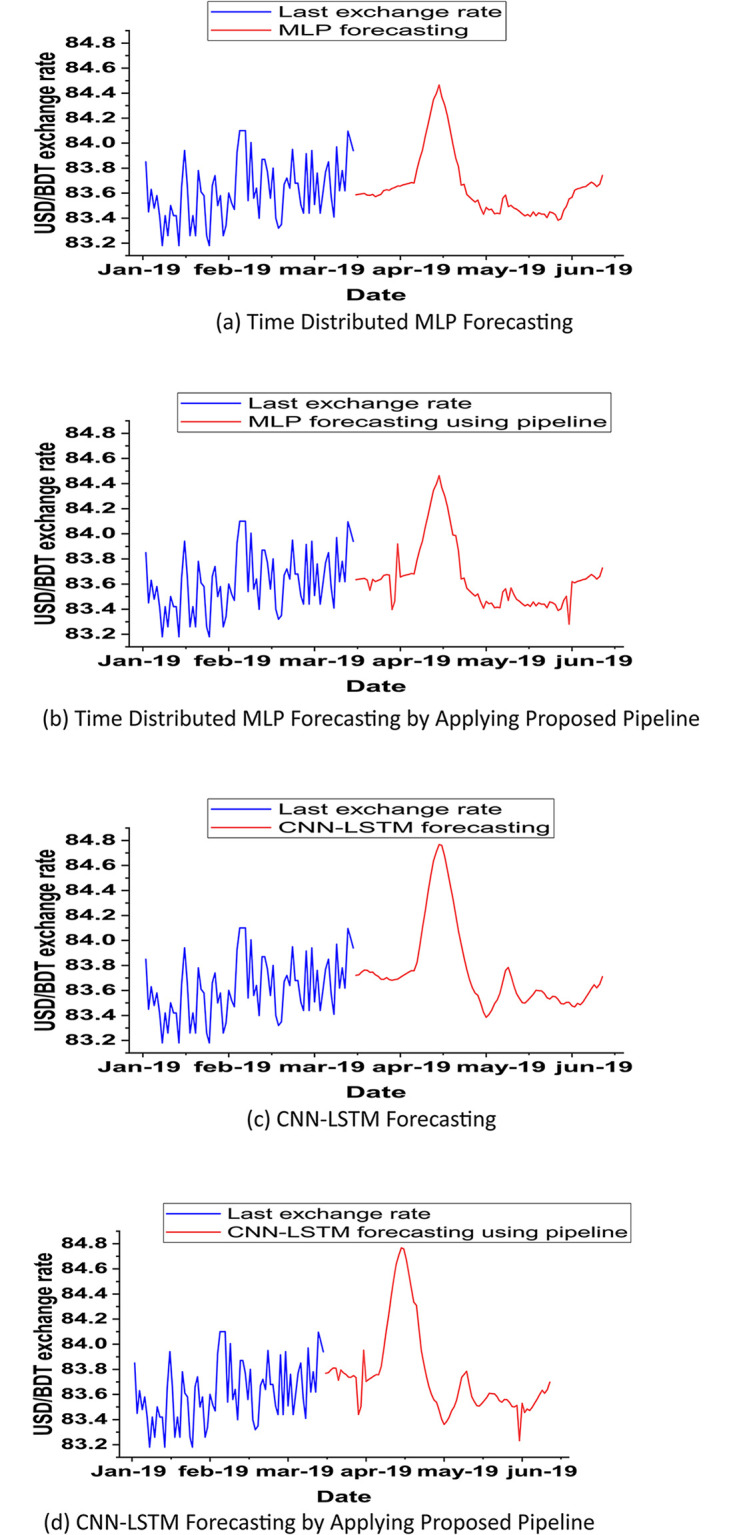
Forecasting the USD/BDT exchange rate by using our best two models. (a) Time distributed MLP, (b) by applying the proposed pipeline on Time Distributed MLP, (c) CNN-LSTM, and (d) by applying the proposed pipeline on CNN-LSTM.

Using our best two models, Time distributed MLP and CNN-LSTM, we have forecasted the exchange rate of USD/BDT for the next 90 days (Fig 3). Then also we applied our proposed model to CNN-LSTM and Time Distributed MLP (Fig 3).

Plotting forecasted price of USD/BDT exchange rate.

## Discussion

After predicting the USD/BDT exchange rate, we have found that Time Distributed MLP provides us with the lowest RMSE of 0.1984, and our second-best model is the CNN-LSTM hybrid model, which provides an RMSE of 0.2059. After applying our proposed pipeline, the RMSE of Time Distributed MLP has been reduced to 0.1900. The RMSE of CNN-LSTM has been reduced to 0.1973 (Table 4). Previously, Mia MS, Rahman MS, Das S [35] Used ANN (Artificial Neural Networks) for predicting the USD/BDT exchange rate, and Alam M, Rahman MS, Mia MS [36] also used ANN for predicting the USD/BDT exchange rate. But in their research, None of them have included any features (Table 5). To compare our research with these latest research works, we have used the same model that these researchers have used for forecasting (Table 5). By using ANN, without applying our proposed pipeline, we have an RMSE of 0.2196, which is better than both researches. Using the same model, we are getting lower RMSE which is a clear sign of improvement. This improvement makes it clear that including the macroeconomics features has provided more relevant information to the model. As a result, our models are getting trained well and performing better than previously trained models. After applying our proposed pipeline to the ANN model, the RMSE has reduced to 0.2189 (Table 5), indicating our proposed pipeline’s effectiveness. For further comparison, we have chosen our best two models. By using Time Distributed MLP, we have the lowest RMSE of 0.1984, and by using the CNN-LSTM model, we have our second-lowest RMSE of 0.2059 (Table 5). Then after applying our proposed pipeline to these two models, the RMSE of Time Distributed MLP has been reduced to 0.1900. The RMSE of CNN-LSTM has been reduced to 0.1973. In both cases, without applying the proposed pipeline and by using the proposed pipeline, we have got better results than previous research. Not only these three models, but also we got better results from Stacked-LSTM, CNN, and Encoder-Decoder without even applying our proposed pipeline to these models. This improvement again demonstrates the effectiveness of using macroeconomic features in our data set. Finally, by applying our proposed pipeline to every model, we have further reduced the RMSE of all the models (Table 4). That illustrates the potential and promising result of our proposed pipeline.

**Table 4:** RMSE of all the models after applying proposed pipeline.

Models	RMSE without applying proposed pipeline	RMSE after applying proposed pipeline
*LSTM*	0.2526	0.2470
*Bi* − *LSTM*	0.2493	0.2372
*Stacked* − *LSTM*	0.2239	0.2234
*GRU*	0.2786	0.2777
*ANN*	0.2196	0.2189
*CNN*	0.2244	0.2159
*CNN* − *LSTM*	0.2059	0.1973
*Encoder* − *Decoder*	0.2257	0.2190
*Time Distributed MLP*	0.1984	0.1900
*SVM*	0.412	0.4056
*XGBoost*	0.2467	0.2366

**Table 5:** Comparing other USD/BDT exchange rate article with our article.

Author	Model	Currency	RMSE
*Mia MS*, *Rahman MS*, *Das S*	ANN	USD/BDT	0.240281
*Alam M*, *Rahman MS*, *Mia MS*	ANN	USD/BDT	0.240
*Our ANN Model*	ANN (without pipeline)	USD/BDT	0.2196
	ANN (applying pipeline)	USD/BDT	0.2189
*Our Best* 2 *Models*	Time distributed MLP(without pipeline)	USD/BDT	0.1984
	Time distributed MLP (applying pipeline)	USD/BDT	0.1900
	CNN-LSTM(without pipeline)	USD/BDT	0.2059
	CNN-LSTM(applying pipeline)	USD/BDT	0.1973

## Conclusion and future work

Forecasting the exchange rate of USD/BDT more accurately can make the FOREX market a secure and reliable place for investors. Previously many researchers tried to forecast the exchange rate of USD/BDT by using time series models, machine learning models, and deep learning models but none of the research included factors that can directly affect the exchange rate of USD/BDT. To introduce a new scope for the researchers, we have included 16 macroeconomic factors in our dataset along with time for training our models. These macroeconomic factors are directly correlated with the USD/BDT exchange rate. Previously Mia MS, Rahman MS, and Das S [35] used Artificial Neural Networks without any factors affecting the USD/BDT exchange rate and got an RMSE of 0.240281. The same work was also done by Alam M, Rahman MS, and Mia MS [36] without any factors, and they got an RMSE of 0.240. In our research, we have got the best RMSE of 0.1984 by using time-distributed MLP and using our proposed pipeline, we have reduced the RMSE of time-distributed MLP to 0.1897. In both cases, we have got better RMSE than previous research works. In the future, research can be conducted using advanced models like transformers with these macroeconomic features to determine if the predictions are even more accurate. This study further creates the scope of rethinking the current exchange rate forecast methodologies, brings in more effective macroeconomic factors, and tests the influence over any exchange rate of a currency pair in the world.
